# Early recurrence, time‐to‐recurrence, and recurrence patterns: Assessing their impact on survival outcomes in head and neck squamous cell carcinoma (R/M‐HNSCC) patients treated with first line platinum‐based chemotherapy

**DOI:** 10.1002/cam4.7047

**Published:** 2024-03-08

**Authors:** Pasvich Pitakpaiboonkul, Chuleeporn Jiarpinitnun, Poompis Pattaranutaporn, Nuttapong Ngamphaiboon

**Affiliations:** ^1^ Division of Medical Oncology, Department of Medicine, Faculty of Medicine, Ramathibodi Hospital Mahidol University Bangkok Thailand; ^2^ Division of Radiation Oncology, Department of Radiology, Faculty of Medicine, Ramathibodi Hospital Mahidol University Bangkok Thailand

**Keywords:** clinical trials, early recurrent head and neck squamous cell carcinoma, pattern of recurrence, R/M‐HNSCC, recurrent/metastatic head and neck squamous cell carcinoma, time to recurrence

## Abstract

**Background:**

R/M‐HNSCC patients typically receive 1L platinum‐based chemotherapy with pembrolizumab or cetuximab. However, the outcomes for patients with early recurrence (<6 months) remain unclear due to their exclusion from most 1L studies. This study aimed to assess the impact of time‐to‐recurrence intervals (TTRI) and recurrence patterns on the survival of R/M‐HNSCC patients.

**Methods:**

We identified non‐curable R/M‐HNSCC patients at our institution from 1/2008 through 6/2020. We analyzed the outcomes of early recurrent patients who received 1L systemic treatment, with different TTRIs and recurrence patterns.

**Results:**

Our study included 234 eligible patients. The majority (47%) experienced early recurrence (<6 months), while 22%, 20%, and 11% had recurrences at 6–12 months, >12 months, and de novo metastasis, respectively. The platinum‐based regimen was the most commonly used chemotherapy (86%), with cetuximab and immunotherapy utilized in 3% and 5% of cases, respectively. Significant differences in PFS and OS were observed among TTRI groups. For patients with early recurrence, both platinum‐doublet and monotherapy treatments significantly improved OS. Locoregional recurrence (47%) was the most common, followed by distant metastasis (22%) and both (20%). Recurrence patterns were significantly associated with OS but not with PFS. In multivariate analysis, TTRI ≥12 months significantly correlated with improved PFS (HR 0.51; *p* = 0.004) and OS (HR 0.58; *p* = 0.009), whereas recurrent pattern did not.

**Conclusion:**

TTRI significantly influenced the survival, while recurrence patterns did not. In our study, the retrospective design limited our ability to definitively establish whether early recurrent R/M‐HNSCC patients would benefit more from platinum‐doublet. Despite poor prognosis, early recurrent patients benefited from 1L systemic treatments. Given the variation in prognoses, TTRI should be considered a stratification factor in future clinical trials.

## 1 BACKGROUND

Head and neck cancer ranks as the seventh most common type of cancer globally,^1^ with approximately 878,348 new cases diagnosed in 2020.^2^ The majority of patients present with locally advanced stages with squamous cell histology.^1^, ^3^ The primary treatment for locally advanced head and neck squamous cell carcinoma (LA‐HNSCC) involves surgery and/or concurrent chemoradiation (CRT).^1^, ^3^, ^4^, ^5^ Cisplatin, a platinum‐based chemotherapy agent, is widely recognized as standard treatment administered concurrently with radiotherapy as a radiosensitizer in both definitive and postoperative treatment.^4^, ^5^ While a minority of patients present with de novo metastasis (M1) at presentation, most patients develop recurrent disease.^1^, ^3^ Recurrence rates for HNSCC patients range between 15% and 65% with locoregional recurrence being the most common pattern.^1^, ^3^, ^5^, ^6^ Cisplatin‐CRT is a standard part of definitive treatment for most patients with LA‐HNSCC.^6^

Patients with recurrent/metastatic HNSCC (R/M‐HNSCC) who are not candidates for curative local therapy generally receive systemic treatment.^4^, ^5^ The current standard treatment for R/M‐HNSCC is platinum‐based chemotherapy combined with pembrolizumab, an immune checkpoint inhibitor (ICI), or cetuximab, an anti‐EGFR monoclonal antibody, depending on programmed death‐ligand 1 (PD‐L1) expression of tumors following the KEYNOTE‐048 study.^4^, ^5^, ^7^ Despite the use of platinum‐doublet chemotherapy combined with ICI or cetuximab, the prognosis for R/M‐HNSCC patients in Phase 3 clinical trials remains poor, with a median overall survival (OS) ranging between 10.7 and 13.9 months.^7^, ^8^, ^9^, ^10^ All Phase 3 randomized studies of first‐line R/M‐HNSCC patients included only those with recurrent disease, either locoregional or distant metastasis, occurring ≥6 months after their last definitive treatment. Patients with recurrent disease <6 months were considered early recurrent HNSCC and were excluded from these Phase 3 studies of first‐line treatment for R/M‐HNSCC.^7^, ^8^, ^9^, ^10^ Furthermore, most early recurrent HNSCC patients had previously received cisplatin‐CRT as part of their definitive treatment and were considered platinum‐resistant due to recurrence <6 months after completing cisplatin‐CRT. Nonetheless, it remains unclear whether a 6‐month interval from the last definitive treatment should be the cutoff for early recurrence and/or platinum‐refractory disease. Current standard guidelines recommend that patients with early or platinum‐refractory R/M‐HNSCC receive monotherapy systemic treatment, either chemotherapy or ICI.^4^, ^5^ These patients are also eligible for most second‐line systemic therapy for platinum‐refractory R/M‐HNSCC clinical trials.^11^, ^12^ However, they received cisplatin monotherapy concurrently with radiotherapy either weekly or every‐3‐week schedule, with the cisplatin dose intended as a radiosensitizer rather than a full platinum‐doublet chemotherapy dose.^13^ Therefore, it is unclear whether patients with early and/or platinum‐refractory R/M‐HNSCC should be offered platinum‐doublet combination or monotherapy with or without ICIs or cetuximab if they are fit for combination chemotherapy. Moreover, the efficacy of first‐line systemic treatment may be affected by the pattern of recurrence.^14^ Locoregional recurrence might represent (chemo‐)radioresistant HNSCC, which may respond differently to systemic treatment compared to distant metastatic disease, as the latter has not been exposed to radiotherapy before.

To the best of our knowledge, only a few retrospective studies have investigated the outcomes of patients with early recurrence, which have shown poor responses to systemic treatment and short survival times.^14^, ^15^, ^16^ Recent clinical trials of first‐line systemic treatment have excluded patients with early recurrent HNSCC.^7^, ^8^, ^9^, ^10^ Therefore, our study aims to evaluate the outcomes of patients with early recurrence who received first‐line systemic treatment. We also sought to determine the appropriate approach to integrate these early recurrent patients into future clinical trial settings for first‐line systemic treatment of R/M‐HNSCC. Furthermore, we assessed the impact of varying time‐to‐recurrence intervals (TTRIs) and patterns of recurrence on the survival of patients with R/M‐HNSCC who received first‐line systemic treatment. Patients with de novo metastasis (M1) at the initial diagnosis were included in this analysis for comparison with patients who had recurrent HNSCC.

## 2 METHODS

### 2.1 Patient and study design

Patients with histologically or cytologically confirmed HNSCC of the oral cavity, oropharynx, larynx, and hypopharynx, treated at the Ramathibodi Cancer Center, Mahidol University from January 2008 through June 2020, were identified via the Ramathibodi Cancer Registry. All eligible patients had to have recurrent or metastatic disease that was not amenable to curative treatment. We retrospectively reviewed the available medical records of these eligible patients for baseline characteristics such as age, sex, Eastern Cooperative Oncology Group (ECOG) performance status, American Joint Committee on Cancer TNM staging (AJCC 8th edition),^17^ smoking status, primary tumor site, histological grade, definitive treatment for initial diagnosis, pattern and time to recurrent disease, and first‐line palliative systemic treatment for R/M‐HNSCC. Exclusion were made for patients with locoregional recurrence who received curative local treatment, such as RT/CRT or radical surgery, as well as for those with primary tumor sites in the nasopharynx or paranasal sinus, unknown primary site, non‐squamous cell histology, secondary primary carcinoma of the head and neck, recurrence more than 5 years after the initial diagnosis of HNSCC, and unavailable medical records. Additionally, patients with persistent disease after definitive RT/CRT who received systemic treatment without evidence of disease progression, or those who progressed during definitive RT/CRT, were excluded. However, patients with persistent disease who demonstrated a response or stable disease following definitive RT/CRT were included in this analysis, only if systemic treatment was started at the time of disease progression. The study was approved by the Ethics Committee of Ramathibodi Hospital, Mahidol University.

### 2.2 Definition of time‐to‐recurrence interval and pattern of R/M‐HNSCC

TTRI was defined as the duration from the date of the last definitive treatment to the date of diagnosis of recurrent disease by imaging or pathological confirmation, which was not amenable to curative treatment. For eligible patients with persistent disease following definitive RT/CRT, TTRI was determined from the date of the last definitive treatment to the date when disease progression was confirmed, either by imaging or pathology. Patients with early R/M‐HNSCC, defined by a TTRI <6 months, based on the exclusion criteria of several major Phase 3 clinical trials.^7^, ^8^, ^9^, ^10^ Patients who had a TTRI of 6 months or longer were categorized into two groups: 6–12 months and >12 months. Patterns of recurrence were categorized into locoregional, distant metastatic, and both. Patients who had distant metastasis at the time of the initial diagnosis of HNSCC were categorized into the de novo metastatic (M1) group.

Progression‐free survival (PFS) was defined as the time from the first day of first‐line systemic therapy for R/M‐HNSCC to the date of imaging confirmed progressive disease or death from any causes, whichever occurred first. OS was defined as the time from the date of diagnosis of R/M‐HNSCC, confirmed by imaging or pathology to death from any cause. The survival status of each patient was cross‐checked with the National Security Death Index of Thailand.

### 2.3 Statistical analyses

The objectives of the study were to evaluate the outcomes of palliative systemic treatment and survival in relation to the TTRI and pattern of recurrence in patients with R/M‐HNSCC. The outcomes of HNSCC patients with early recurrence were also explored comparing with R/M‐HNSCC patients who had TTRI ≥6 months, following major clinical trial exclusion criteria for first‐line systemic treatment.^7^, ^8^, ^9^, ^10^ R/M‐HNSCC patients who received first‐line palliative systemic treatment were evaluated for both PFS and OS, whereas patients who did not receive such systemic treatment were evaluated for OS only. Descriptive statistics were used to describe baseline patient characteristics and treatment. Categorical variables were presented as numerals and percentages. Differences between the categorial data were compared using the Fisher's exact test or chi‐squared test as appropriate. Continuous variables were expressed as mean, standard deviation (SD), median, and interquartile range. PFS and OS with 95% confidence intervals (CI), were estimated by the Kaplan–Meier method and compared by the log‐rank test. Univariate and multivariate analyses were performed using Cox regression analysis with a level of significance of <0.05. All statistical analyses were conducted using Stata software version 16.0.

## 3 RESULTS

### 3.1 Patient characteristics and palliative systemic treatment in R/M‐HNSCC patients

A total of 1281 patients with HNSCC were identified. Patients were excluded for no recurrent or metastatic disease (*N* = 894), second primary HNSCC (*N* = 31), and loss of follow‐up (*N* = 95). Additionally, out of 137 patients with locoregional recurrence, 27 were excluded from the study because they underwent curative local treatment. A total of 234 patients were eligible for analysis (Figure S1). Baseline characteristics of R/M‐HNSCC by TTRI are summarized in Table 1. Patients with de novo metastasis (M1) had significantly more advanced T (*p* = 0.002) and N (*p* = 0.002) stages at initial diagnosis and higher poorly differentiated histology (*p* = 0.034). Patients who had a TTRI >12 months received significantly more adjuvant radiotherapy (RT) when compared to the other groups (*p* = 0.047). There were no statistically significant differences in baseline patient characteristics such as age at initial diagnosis, sex, smoking status, primary site, previous curative surgery, and pattern of recurrence. Cisplatin was the major regimen for CRT in all groups. The median TTRI was 3 months (1.8–4.5 months) in <6 months group, 7.6 months (6.6–9.6 months) in 6–12 months group, and 17.6 months (14.7–27.4 months) in >12 months group. Locoregional recurrence was the most common pattern of recurrence in every recurrent group.

**TABLE 1:** Characteristics and treatments of patients with R/M‐HNSCC.

Characteristics	<6 months N = 109 (%)	6–12 months N = 52 (%)	>12 months N = 47 (%)	De novo metastasis N = 26 (%)	*p*‐value
Characteristics at initial diagnosis					
Median age (range)	61 (54–70)	58 (49–69)	64 (57–71)	56 (52–64)	0.078
Age ≥65 years	38 (35)	18 (35)	21 (45)	6 (23)	0.318
Sex (*N*, %)					
Male	86 (79)	46 (89)	38 (81)	20 (77)	0.477
Female	23 (21)	6 (11)	9 (19)	6 (23)	
Smoking status					
Ever	70 (68)	35 (76)	33 (77)	20 (77)	0.572
Missing	6	6	4	0	
Primary site					
Oral cavity	46 (42)	16 (31)	19 (40)	2 (8)	0.079
Oropharynx	28 (26)	18 (35)	8 (17)	9 (34)	
p16/HPV positive	3 (11)	3 (17)	2 (25)	1 (11)	
p16 and HPV negative	12 (43)	10 (56)	0	5 (56)	
Larynx	16 (15)	8 (15)	9 (19)	6 (24)	
Hypopharynx	19 (17)	10 (19)	11 (24)	9 (34)	
Histology type					
Well differentiation	30 (28)	9 (17)	13 (27)	2 (8)	0.034
Moderate differentiation	57 (52)	32 (61)	18 (38)	11 (42)	
Poorly differentiation	2 (2)	4 (8)	4 (9)	4 (15)	
Undifferentiation	6 (6)	4 (8)	4 (9)	2 (8)	
No specified	14 (12)	3 (6)	8 (17)	7 (27)	
Clinical T stage at diagnosis					
T1‐2	23 (21)	23 (44)	14 (30)	2 (8)	0.002
T3‐4	86 (79)	29 (56)	32 (70)	24 (92)	
Clinical N stage at diagnosis					
N0‐1	53 (49)	15 (29)	22 (47)	3 (11)	0.002
N2	38 (35)	27 (52)	21 (45)	13 (50)	
N3	18 (16)	10 (19)	4 (8)	10 (39)	
Previous curative surgery	51 (47)	17 (33)	24 (51)	N/A	0.136
Previous adjuvant RT	41 (38)	12 (23)	20 (43)	N/A	0.047
CRT	30 (73)	9 (75)	8 (42)		
RT alone	11 (27)	3 (25)	11 (58)		
Previous definite RT	58 (53)	35 (67)	23 (49)	N/A	0.743
CRT	55 (95)	34 (97)	21 (91)		
RT alone	3 (5)	1 (3)	2 (9)		
Previous CRT	82 (75)	42 (81)	28 (60)	N/A	0.204
Cisplatin	55 (67)	34 (81)	18 (64)		
Non cisplatin	27 (33)	8 (19)	10 (36)		
Missing	3	1	2		
Median time from last definitive treatment to recurrence (months, IQR)	3 (1.8–4.5)	7.6 (6.6–9.6)	17.6 (14.7–27.4)	N/A	<0.001
Pattern of recurrence					
Locoregional	55 (51)	20 (38)	24 (51)	N/A	0.322
Distant	30 (27)	18 (35)	17 (36)		
Both	24 (22)	14 (27)	6 (13)		
Systemic treatment of RM‐HNSCC patients					
Treatment regimen (N, %)					
Platinum base	61 (56)	35 (67)	25 (53)	13 (50)	0.473
Chemotherapy	58 (53)	34 (65)	18 (38)	13 (52)	
Chemotherapy + Anti‐EGFR	3 (3)	1 (2)	3 (6)	0	
Non‐platinum base chemotherapy	7 (6)	2 (4)	4 (9)	1 (4)	
Immunotherapy alone	1 (1)	2 (4)	3 (6)	1 (4)	
ICI + Platinum based chemotherapy	0	0	4(9)	0	
No treatment	40 (37)	13 (25)	15 (32)	11 (42)	
Number of agents					
Combination	61 (88.4)	35 (89.7)	25 (78.1)	13 (86.7)	0.508
Single agent	8 (11.6)	4 (10.3)	7 (21.9)	2 (13.3)	

Among R/M‐HNSCC patients who received first‐line systemic treatment, the platinum‐based regimen was the most common chemotherapy regimen (86%) used in first‐line palliative treatment and not differ significantly among each group (Table 1). Most patients were treated with combination chemotherapy. The most common non‐platinum based chemotherapy was paclitaxel monotherapy (5.1%). In this study, cetuximab and ICI were utilized in 7 (3%) and 11 (5%) patients, respectively. Only TTRI >12 months group had chemotherapy combined with immunotherapy (9%). The percentages of patients who did not receive systemic treatment were 37%, 25%, 32%, and 42%, respectively.

### 3.2 Time to recurrence interval and survivals

**FIGURE 1:**
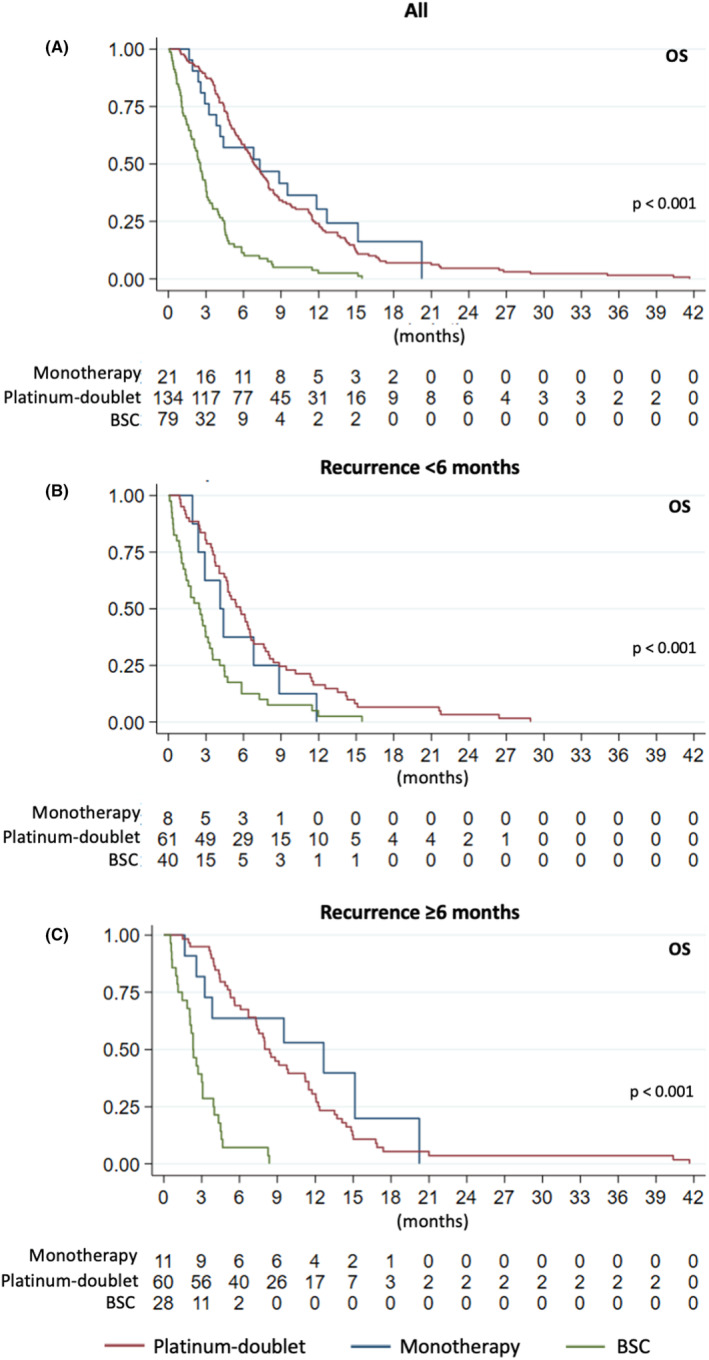
OS of R/M HNSCC patients who received 1L platinum‐doublet chemotherapy, monotherapy (including ICI), and best supportive care (BSC) and time to recurrence interval (TTRI).

**FIGURE 2:**
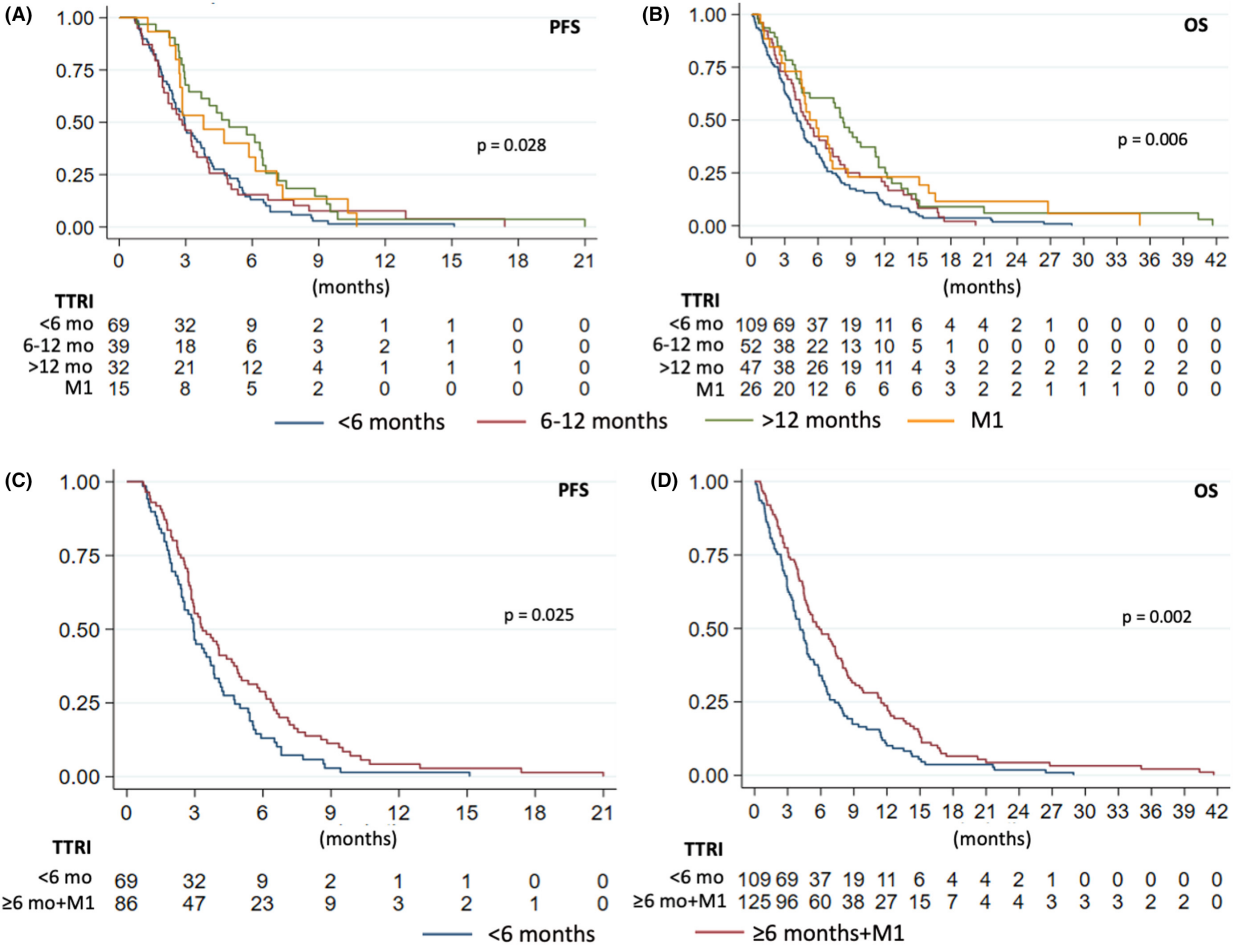
PFS and OS of R/M‐HNSCC patients by time to recurrence interval (TTRI).

The median follow‐up duration of the study was 6.9 months. The median PFS and OS for all R/M‐HNSCC patients were 3.2 and 4.8 months, respectively (Figures S2 and S3). For R/M‐HNSCC patients who received first‐line systemic treatment, the median OS of patients who received platinum‐doublet chemotherapy, monotherapy (including ICI), and best supportive care (BSC) were 6.9, 7.3, and 2.6 months (*p* < 0.001), respectively (Figure 1A). In all recurrent HNSCC patients previously treated with cisplatin‐based CRT, platinum‐based chemotherapy significantly prolonged the median OS compared to non‐platinum based chemotherapy and BSC (6.6 vs. 4.4 vs. 1.6 months; *p* < 0.001). (Figure S4) In patients with early recurrence (<6 months), those who received platinum‐based doublet and monotherapy chemotherapy had significantly longer OS when compared with BSC (5.8 vs. 4.1 vs. 2.5 months; *p* < 0.001) (Figure 1B). Similarly, for patients with a TTRI ≥6 months, the median OS for those who received platinum‐based doublet, monotherapy chemotherapy, and BSC were 8.4, 12.7, and 2.3 months (*p* < 0.001), respectively (Figure 1C). There was no statistically significant correlation between TTRI and OS of R/M‐HNSCC patients (*r* ^2^ = 0.14; *p* = 0.051), but a trend was observed (Figure S5A). The majority of patients in this study had early recurrent HNSCC (47%), whereas 52 (22%) and 47 (20%) patients had TTRI 6–12 and >12 months, respectively. There were 26 patients (11%) with de novo metastatic (M1) HNSCC. The OS of early relapsed patients (TTRI <6 months) previously treated with RT alone or CRT did not show a significant difference (4.1 vs. 4.6 months; *p* = 0.964). However, for patients who relapsed with a TTRI ≥6 months, those who had received prior CRT demonstrated a significantly longer OS compared to those treated with RT alone (7.4 vs. 4.1 months; *p* = 0.017) (Figure S6). There was a significant difference in PFS (*p* = 0.028) and OS (*p* = 0.006) between each group. The median PFS of early recurrence, TTRI 6–12, >12 months, and M1 were 2.9, 2.8, 5.0, and 3.8, respectively (Figure 2A). The median OS of early recurrence, TTRI 6–12, >12 months, and M1 were 4.1, 5.1, 8.3, and 5.3, respectively (Figure 2B).

Using major clinical trial eligibility cutoffs, patients with early recurrence (<6 months) had significantly worse survival outcomes than those with TTRI ≥6 months and the M1 group for both PFS and OS (Figure 2C,D). The median PFS was 2.9 vs. 3.3 months (HR 0.69 [95% CI 0.50–0.96; *p* = 0.025]). The median OS was 4.1 vs. 5.9 months (HR 0.66 [95% CI 0.51–0.87; *p* = 0.002]). Furthermore, when using a 12‐month cutoff, the PFS and OS in the TTRI <12 months group had significantly worse survival outcomes than those at ≥12 months and the M1 group (Figure S7 and S8). The median PFS was 2.9 vs. 4.6 months [HR 0.59 (0.41–0.84, *p* = 0.003)]. The median OS was 4.5 vs 7.1 months (HR 0.62 [0.47–0.84, *p* = 0.002]). In the multivariate analysis, TTRI >12 months was significantly correlated with both PFS (HR 0.51 [95% CI 0.32–0.81]; *p* = 0.004) and OS (HR 0.58 [95% CI 0.39–0.87]; *p* = 0.009) (Table 2).

**TABLE 2:** Univariate and multivariate analyses for PFS and OS of R/M‐HNSCC patients.

Factors	PFS				OS			
	Univariate		Multivariate		Univariate		Multivariate	
	OR (95%CI)	*p*‐value	OR (95%CI)	*p*‐value	OR (95%CI)	*p*‐value	OR (95%CI)	*p*‐value
Age: <65 vs. ≥65	0.83 (0.59–1.18)	0.3			1.04 (0.79–1.37)	0.766		
Sex: Female vs. male	0.83 (0.55–1.27)	0.395			0.80 (0.57–1.12)	0.185		
Smoking: Never vs. ever	0.73 (0.50–1.06)	0.099			0.74 (0.55–1.01)	0.056		
Primary: Non‐oropharynx vs. oropharynx	0.84 (0.60–1.20)	0.344	0.81 (0.54–1.22)	0.312	0.72 (0.54–0.97)	0.032	0.86 (0.59–1.24)	0.418
Definite treatment: Surgery vs. CRT	0.51 (0.35–0.73)	<0.001	0.55 (0.37–0.82)	0.003	0.66 (0.49–0.90)	0.008	0.77 (0.54–1.10)	0.146
CRT regimen: Cisplatin vs. non‐cisplatin	0.98 (0.64–1.51)	0.933			0.92 (0.64–1.32)	0.665		
TTRI								
<6 months	Ref		Ref		Ref		Ref	
6–12 months	0.91 (0.61–1.36)	0.644	1.05 (0.69–1.59)	0.832	0.81 (0.58–1.13)	0.214	0.85 (0.58–1.26)	0.417
>12 months	0.54 (0.35–0.84)	0.006	0.51 (0.32–0.81)	0.004	0.56 (0.39–0.80)	0.002	0.58 (0.39–0.87)	0.009
De novo metastasis	0.64 (0.37–1.13)	0.127			0.63 (0.40–0.97)	0.037		
Recurrence vs. De novo metastasis	0.78 (0.46–1.34)	0.368			0.77 (0.50–1.17)	0.215		
Pattern of recurrence								
Locoregional	Ref		Ref		Ref		Ref	
Distant metastasis	1.06 (0.72–1.58)	0.765	0.85 (0.56–1.29)	0.441	0.85 (0.62–1.16)	0.309	0.80 (0.56–1.16)	0.242
Both	1.11 (0.71–1.75)	0.642	0.96 (0.60–1.54)	0.859	1.51 (1.05–2.19)	0.028	1.46 (0.96–2.21)	0.076
1L: Single vs. combination	0.81 (0.51–1.31)	0.394			1.11 (0.67–1.84)	0.698		
1L: Platinum vs. non platinum	1.22 (0.77–1.97)	0.394			0.90 (0.54–1.50)	0.698		

### 3.3 Pattern of recurrence and survivals

Locoregional recurrence was the most common (47%), whereas the distant metastasis only accounted for 22.2%. (Figure S5B) There were 47 patients (20%) and 26 patients (11%) had both locoregional and distant metastatic recurrence, and de novo metastasis (M1) at the initial diagnosis, respectively. The mean time to recurrence in the locoregional recurrence group was 8.6 months, while distant metastasis and both groups were 9.4 and 7.4 months, respectively (Figure S5C).

**FIGURE 3:**
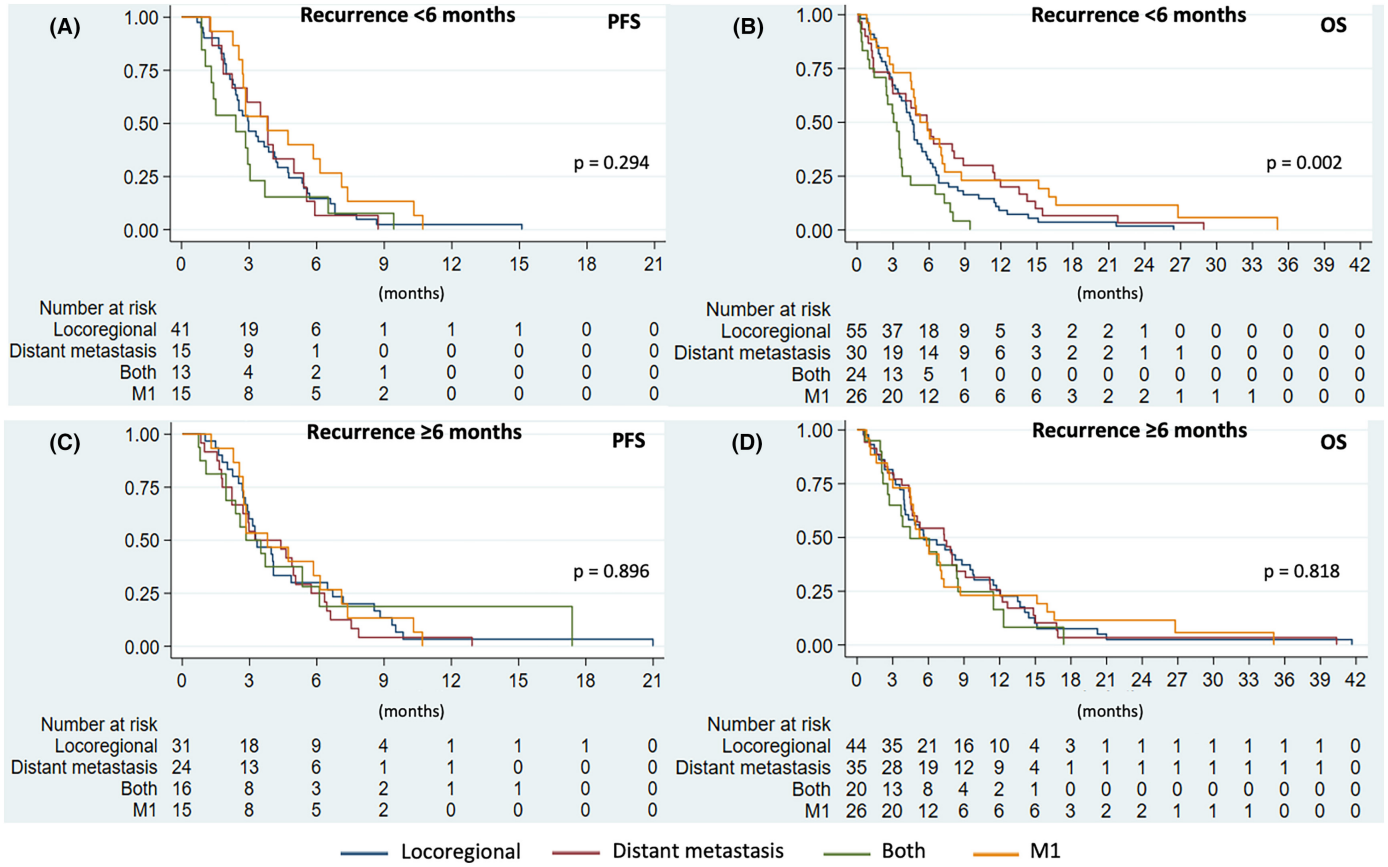
PFS (A) and OS (B) in group TTRI <6 months and De novo Metastasis; PFS (C) and OS (D) in group TTRI ≥6 months and M1.

There was no significant difference in PFS among each pattern of R/M‐HNSCC (*p* = 0.790). However, the different pattern of R/M‐HNSCC was significantly associated with differences in OS (*p* = 0.019). The median OS of locoregional, distant, both, and M1 were 4.8, 6.2, 3.6, and 5.3, respectively. In patients with early recurrence (<6 months) compared with the M1 group, the pattern of recurrence was not significantly associated with PFS (*p* = 0.294), but with OS (*p* = 0.002) (Figure 3A,B). The median OS of the early R/M‐HNSCC group, categorized by locoregional, distant, both, and M1, were 4.6, 5.9, 3.3, and 5.3, respectively. On the other hand, there was no significant difference in both PFS and OS by pattern of recurrence in R/M‐HNSCC patients who recurred after 6 months (Figure 3C,D). In the multivariate analysis, R/M‐HNSCC patients with both locoregional and distant metastatic recurrence did not show a significant association with either PFS or OS compared to patients with only locoregional recurrence (Table 2). Furthermore, patients with a TTRI >12 months had significantly better PFS (HR 0.51 [95% CI 0.32–0.81]; *p* = 0.004) and OS (HR 0.58 [95% CI 0.39–0.87]; *p* = 0.009) compared to those with a TTRI of less than 6 months.

## 4 DISCUSSION

In our study, the survival outcomes of patients with R/M‐HNSCC were shorter compared to the OS reported in Phase 3 clinical trials of first‐line systemic treatment for R/M‐HNSCC.^7^, ^8^, ^9^, ^10^ However, the survival rates observed in our study were comparable to those reported in several real‐world studies of R/M‐HNSCC patients.^18^, ^19^, ^20^ The variation in survival outcomes can be attributed to differences in ethnicity, patient characteristics, primary tumor site, the low incidence of HPV‐related HNSCC in Thailand, different healthcare policies, and variations in clinical practices across countries.^20^, ^21^, ^22^ While ICIs and cetuximab are considered standard of care for R/M‐HNSCC patients in several clinical practice guidelines,^4^, ^5^ only a small number of patients in our cohort received ICI or cetuximab due to limited reimbursement under the healthcare policy in Thailand. This factor could, to some extent, have influenced the observed survival outcomes in our study. Although the study cohort is unique in today's era, as few patients had access to immunotherapy which is now standard of care for recurrent/metastatic disease.^4^, ^5^ However, the study still provides insights into patient prognosis for those who cannot receive immunotherapy. This remains an important discussion for which limited data exists in the real‐world. The study shows that PFS was relatively short, but still better than best supportive care, even when having failed prior platinum‐based chemotherapy as part of treatment for locally advanced disease.

In our study, we observed poor survival outcomes in patients with early recurrent HNSCC (TTRI <6 months), which was consistent with previous studies.^14^, ^15^, ^16^ Notably, current clinical trials investigating first‐line systemic treatment in R/M‐HNSCC typically exclude these patients. Our findings suggest that early recurrent patients benefited from systemic treatment, including both monotherapy (including ICI) and platinum‐doublet chemotherapy, compared to BSC. Among R/M‐HNSCC patients with a TTRI ≥6 months, who met the inclusion criteria for most first‐line systemic treatment clinical trials, the real‐world OS observed in our study was also comparable to the previous reports.^18^, ^19^, ^20^ Importantly, different TTRI cutoff points of 6 and 12 months in our study yielded distinct survival outcomes among R/M‐HNSCC patients. This observation is consistent with a nationwide registry study in Taiwan, which observed varying survival outcomes in patients with relapsed oral cavity squamous cell carcinoma using a TTRI cutoff of 330 days.^19^ These findings potentially support the incorporation of TTRI as a stratification factor in future clinical trials investigating first‐line systemic treatment for R/M‐HNSCC.

The impact of the pattern of recurrence in patients with TTRI ≥6 months on survival outcomes was not significantly different in our study. This finding was consistent with the results from the KEYNOTE‐048 study, a pivotal, randomized, open‐label, Phase 3 clinical trial of pembrolizumab alone or in combination with platinum‐based chemotherapy as first‐line systemic treatment for patients with R/M‐HNSCC.^7^ In the KEYNOTE‐048 sub‐analysis study, no difference was observed in the benefits of 1L systemic treatment for HNSCC patients with either metastatic‐only or locoregional recurrence in both PFS and OS.^23^ However, It should be noted that patients with early recurrence were not included in the KEYNOTE‐048 study. Interestingly, in our study, the pattern of recurrence was significantly associated with OS in patients with early recurrence. The outcomes of patients with distant metastasis only were comparable to those with de novo metastasis. This finding may be partly attributed to the possibility that distant recurrent tumors were not exposed to the full dose of platinum‐doublet chemotherapy during previous treatment with (chemo)radiotherapy. These patients received cisplatin monotherapy concurrently with radiotherapy, following either a weekly or every‐3‐week schedule, where the cisplatin dose was intended as a radiosensitizer.^4^, ^5^, ^13^ As a result, locoregional recurrence could indicate (chemo)radioresistant HNSCC, which might exhibit a different response to systemic treatment compared to distant metastatic disease.

In this study, we reported survival outcomes for R/M‐HNSCC patients experiencing early recurrence, a population typically excluded from first‐line systemic treatment clinical trials.^7^, ^8^, ^9^, ^10^ As the OS of early recurrent patients and those with TTRI ≥6 months demonstrated significant differences, these findings support the possibility that future clinical trials might continue to exclude patients with early recurrence due to their distinct prognosis. However, it is important to note that that the majority of R/M‐HNSCC patients (47%) in our study had early recurrence and still derived benefits from systemic treatment. A potential alternative for future clinical trials could consider including early recurrent patients with appropriate stratification in the study design. Implementing this strategy may not only expedite patient enrollment in the studies but also provide valuable insights into treatment outcomes for this specific patient population.

However, this study has some limitations due to its retrospective nature. The systemic treatments in this study varied in regimens, dosages, and schedules. Additionally, the tolerability of each regimen was not described, a limitation also attributed to the study's retrospective design. This aspect underscores the need for cautious interpretation of the results, as the lack of uniformity in treatment approaches and the absence of comprehensive tolerability data may influence the outcomes and their generalizability. The choice of systemic treatment was determined by physician discretion and involved discussion with the patient and their family members. Access to ICIs and cetuximab was limited for patients in this study due to reimbursement issues stemming from healthcare policy in Thailand. Furthermore, the study couldn't definitively determine if R/M‐HNSCC patients, especially those previously treated with cisplatin‐based chemoradiotherapy in each TTRI, would benefit more from platinum‐doublet chemotherapy or monotherapy (including ICIs and cetuximab). This is due to the relatively small number of patients receiving monotherapy as a first‐line systemic treatment and potential selection bias inherent in the study's retrospective nature. Future prospective randomized studies are necessary to evaluate these factors. However, our retrospective study did not demonstrate a benefit of platinum‐doublet chemotherapy for early recurrent patients. Therefore, future prospective studies should focus on ICIs and cetuximab, with or without platinum‐based chemotherapy, for patients with early recurrence.

## 5 CONCLUSION

In our study, the time to recurrence interval significantly impacted the survival of R/M‐HNSCC patients, whereas the pattern of recurrence did not. Despite a poor prognosis, HNSCC patients with early recurrence (<6 months) may benefit from systemic treatment, either monotherapy or platinum‐based doublet chemotherapy. However, the retrospective design limited our ability to definitively establish whether early recurrent R/M‐HNSCC patients would benefit more from platinum‐doublet chemotherapy. Although most Phase 3 clinical trials for first‐line systemic treatment for R/M‐HNSCC exclude patients with early recurrence, which represented the vast majority of R/M‐HNSCC patients in our study, patients with a time to recurrence of >12 months and de novo metastasis may have different prognoses compared to patients with a time to recurrence of 6–12 months and early recurrence. Therefore, future clinical trials for first‐line systemic treatment of R/M‐HNSCC should consider the time to recurrence interval as a potential stratification factor.

## AUTHOR CONTRIBUTIONS

**Pasvich Pitakpaiboonkul:** Conceptualization (equal); data curation (equal); formal analysis (lead); investigation (equal); methodology (equal); validation (lead); writing – original draft (lead). **Chuleeporn Jiarpinitnun:** Data curation (equal); validation (equal); writing – review and editing (supporting). **Poompis Pattaranutaporn:** Software (lead); visualization (supporting); writing – review and editing (supporting). **Nuttapong Ngamphaiboon:** Conceptualization (equal); data curation (supporting); formal analysis (supporting); funding acquisition (lead); investigation (supporting); methodology (supporting); resources (lead); supervision (lead); writing – original draft (supporting); writing – review and editing (lead).

## FUNDING INFORMATION

The study was funded by the Mahidol University through the Government Research Grant #3484 (Project #6908), Health Systems Research Institute (HSRI) (grant number 63–140), and National Science Research Fund (NSRF) via the Program Management Unit for Human Resources & Institutional Development, Research and Innovation (grant number B05F650041).

## CONFLICT OF INTEREST STATEMENT

N. Ngamphaiboon reports institutional research funding from Pfizer, MSD, Roche, RAPT therapeutics, BeiGene, and Boehringer Ingelheim Pharmaceuticals; and personal fees and nonfinancial support from MSD, Roche, Merck, Eisai, BMS, BeiGene, and Ascendant Biotech Corporation. The other authors declare no conflict of interest.

## ETHICS STATEMENT

This study was approved by the Ramathibodi Ethics Committee of Ramathibodi Hospital, Mahidol University, Bangkok, Thailand. The requirement for informed consent was waived by the Committee due to the study's retrospective chart review nature.

## DECLARATION OF GENERATIVE AI AND AI‐ASSISTED TECHNOLOGIES IN THE WRITING PROCESS

During the preparation of this work the author used ChatGPT (GPT‐4) solely to enhance readability and correct grammar. ChatGPT was not involved in any other conceptual framework aspects of the work, such as study design, data analysis, interpretation, reference sourcing, or manuscript drafting. After using ChatGPT, the authors reviewed and edited the content as needed and take full responsibility for the content of the publication.

## Supporting information


Data S1.

## Data Availability

The data that support the findings of this study are available from the corresponding author upon reasonable request.
