# Supplementary material for: Early recurrence, time‐to‐recurrence, and recurrence patterns: Assessing their impact on survival outcomes in head and neck squamous cell carcinoma (R/M‐HNSCC) patients treated with first line platinum‐based chemotherapy
Source: Cancer Med. 2024 Mar 8;13(4):e7047. doi: 10.1002/cam4.7047 (PMC10922020; doi:10.1002/cam4.7047)
Supplement: Supplementary file 1 — Data S1. [file CAM4-13-e7047-s001.pdf]

Supplement 1: Consort diagram

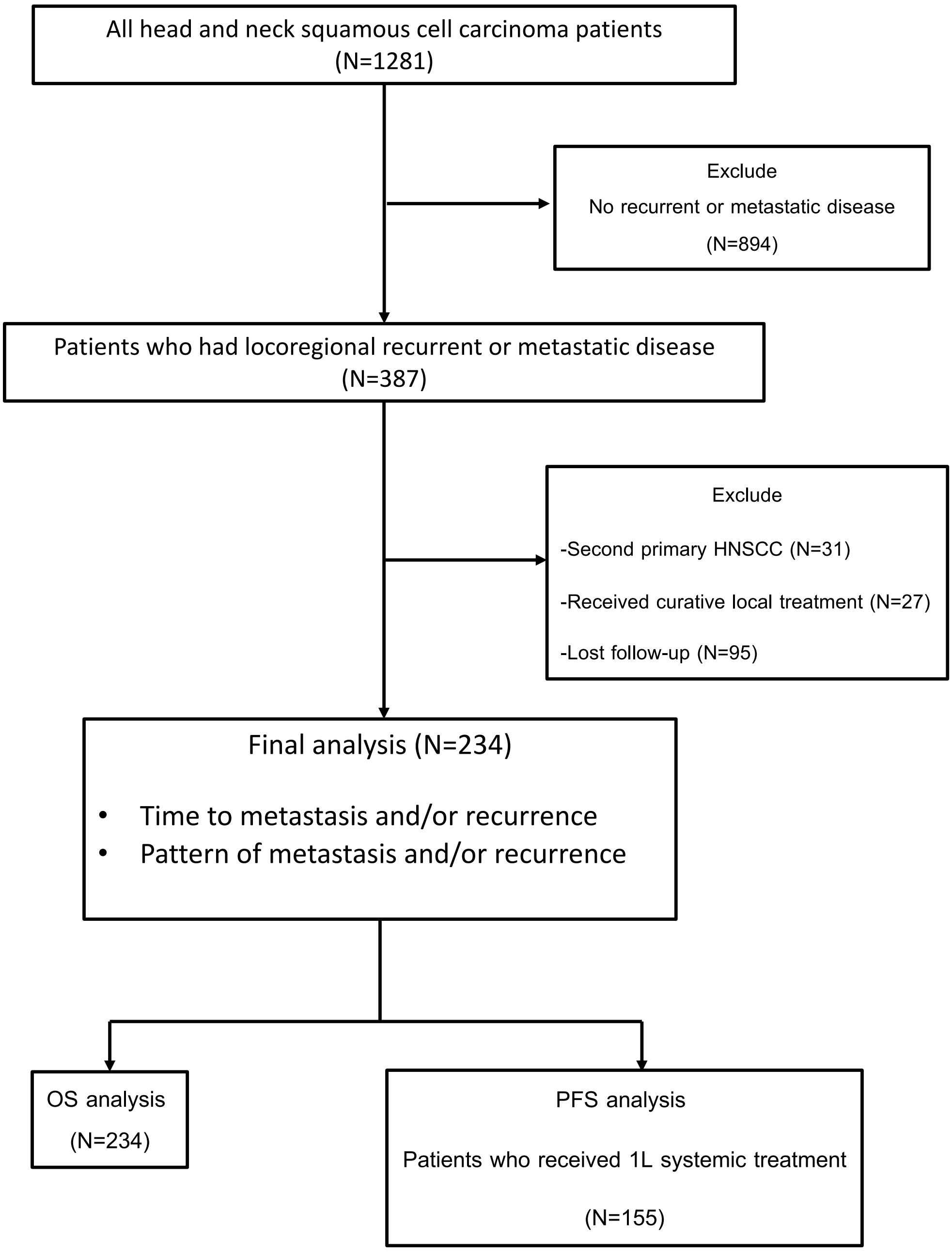

Supplement 2: PFS of all RM-HNSCC patients

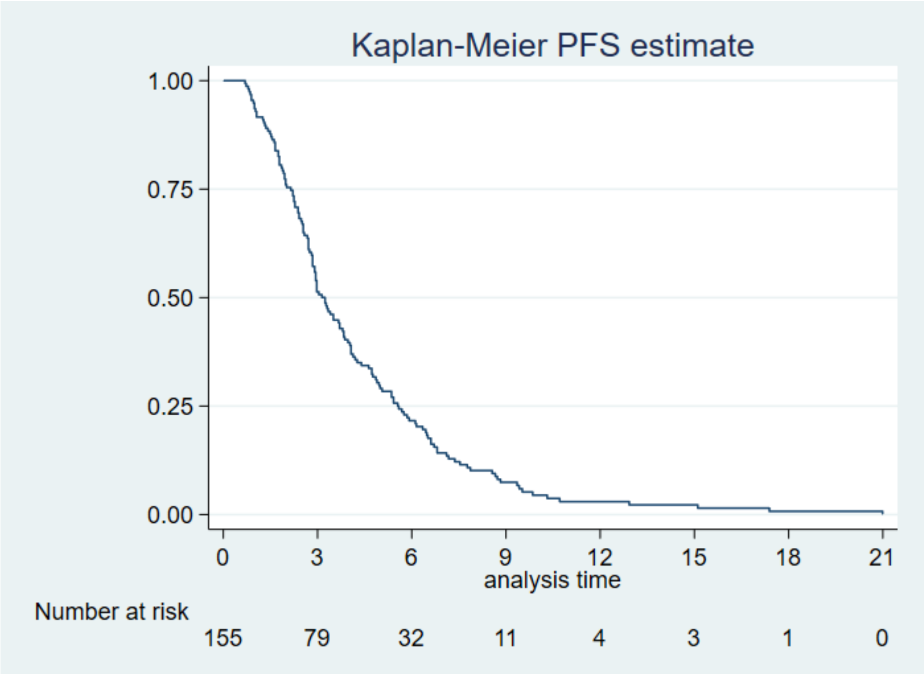

Median PFS = 3.2 (2.8-3.7) months

Supplement 3: OS of all RM-HNSCC patients

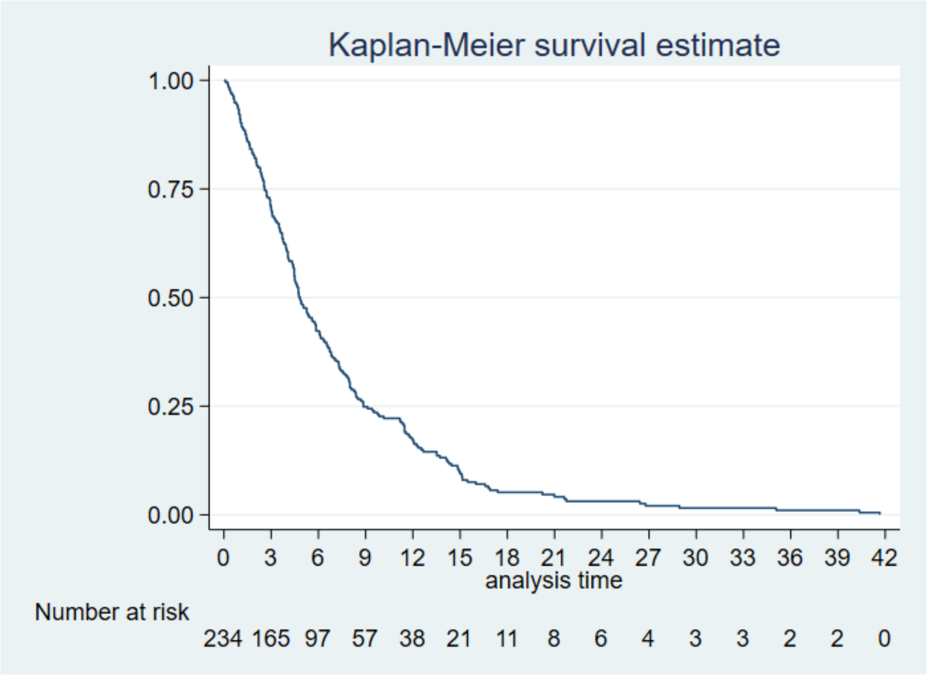

Median OS = 4.8 (4.5-5.8) months

Supplement 4: OS of RM-HNSCC patients who previously received cisplatin-based CCRT with 1L systemic treatment by type of regimen.

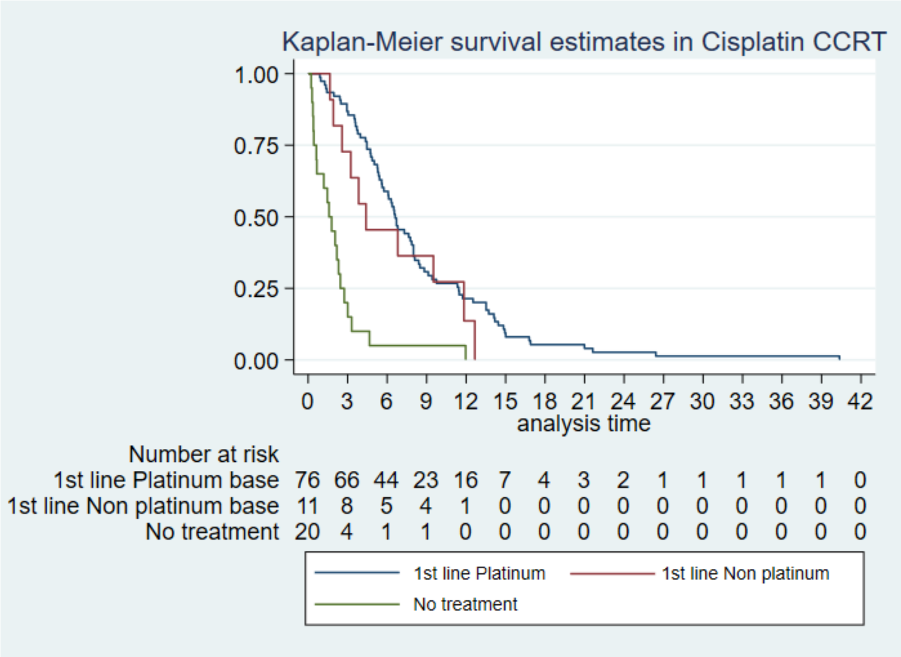

Median OS of 1st line Platinum = 6.6 (5.6-7.9) months

Median OS of 1st line Non platinum = 4.4 (1.9-11.3) months

Median OS of No treatment = 1.6 (0.4-2.3) months

P value <0.001

Supplement 5 Scatter plot of time to recurrence interval (TTRI) and OS of RM-HNSCC patients (A); Pattern of recurrence (B); Duration of last definitive treatment to recurrence by pattern of recurrence (C)

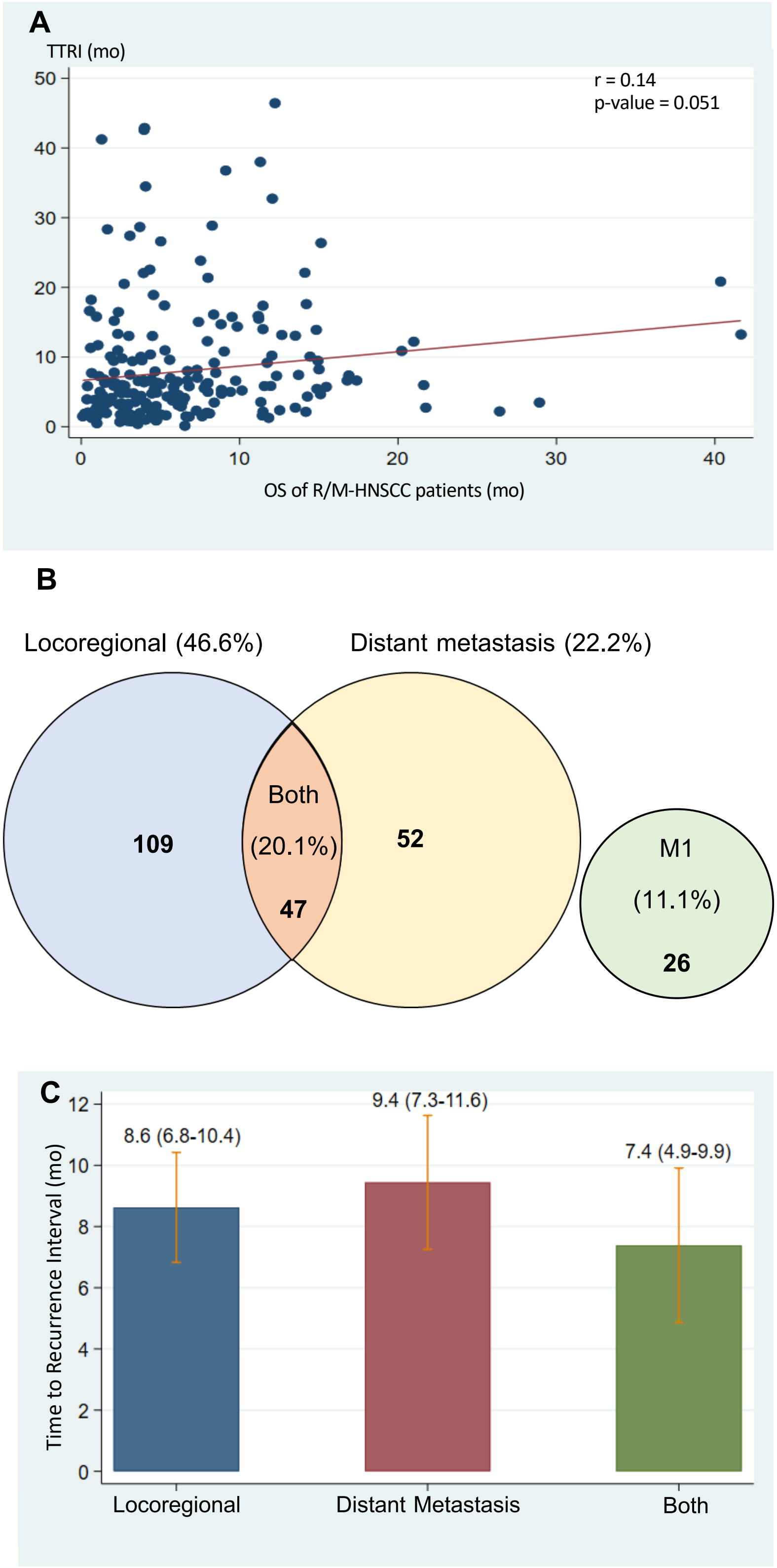

Supplement 6. Impact of TTRI on OS for R/M-HNSCC patients who were previously treated with RT alone or CRT.

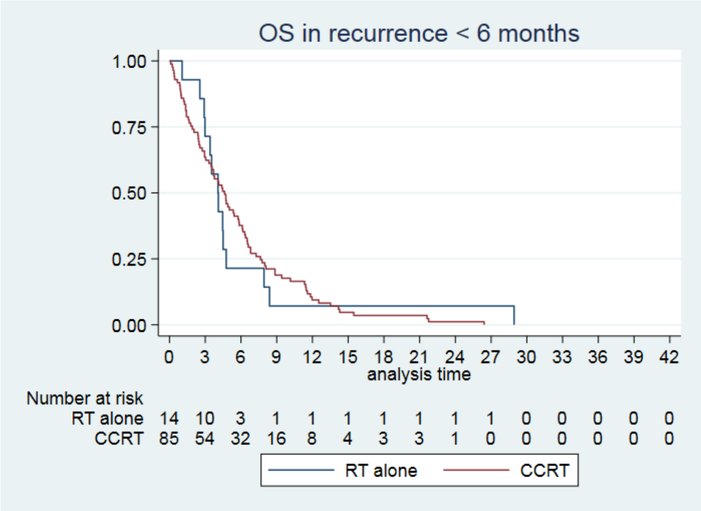

CRT = 4.6 (3.3-5.8) months

RT alone = 4.1 (2.9-4.8) months

HR 1.01 (0.56-1.83), p =0.964

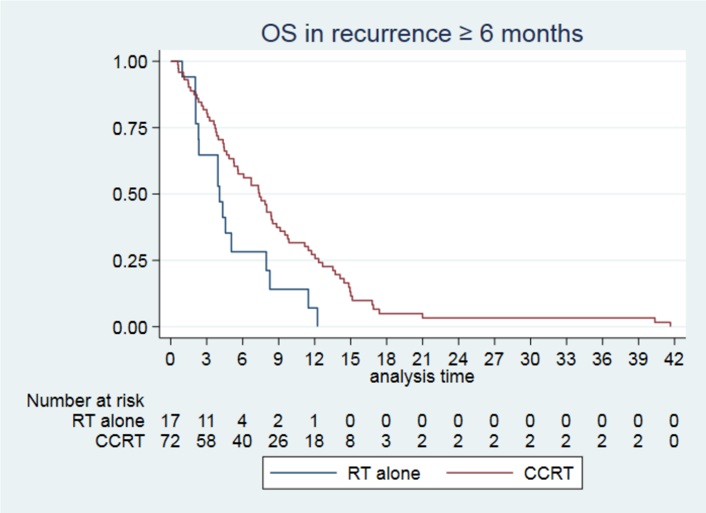

CRT = 7.4 (5.3-8.8) months

RT alone = 4.1 (2.1-8.0) months

HR = 0.48 (0.27-0.84), p=0.017

Supplement 7. PFS of RM-HNSCC patients by duration of recurrent and metastatic disease using TTRI 12 months as a cut-off.

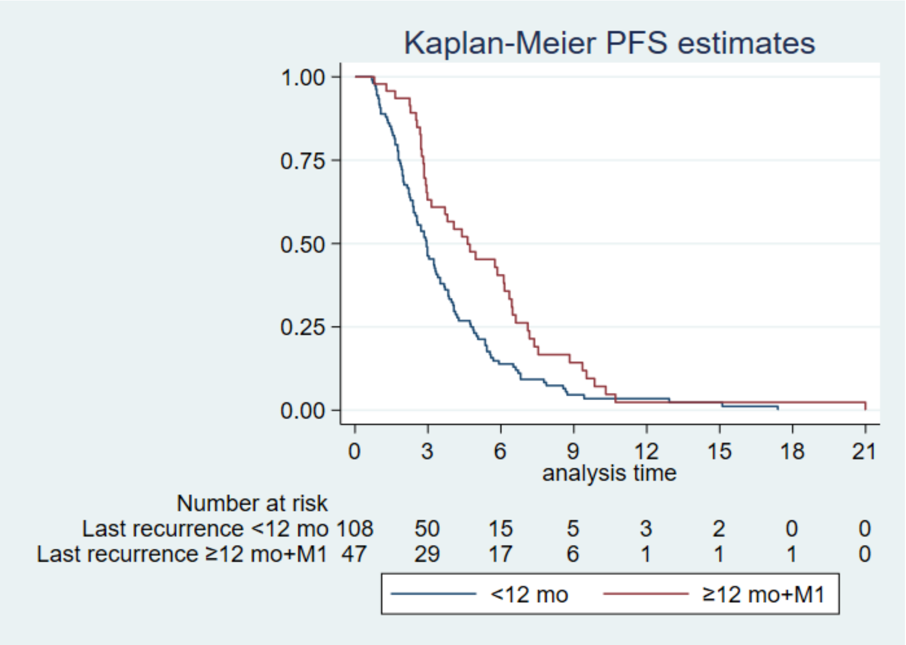

Median PFS TTRI <12 mo = 2.9 (2.4-3.3) months

Median PFS TTRI ≥12 mo + M1 = 4.6 (2.9-6.1) months

HR = 0.59 (0.41-0.84), p-value = 0.003

Supplement 8 OS RM-HNSCC patients by duration of recurrent and metastatic disease using TTRI 12 months as a cut-off.

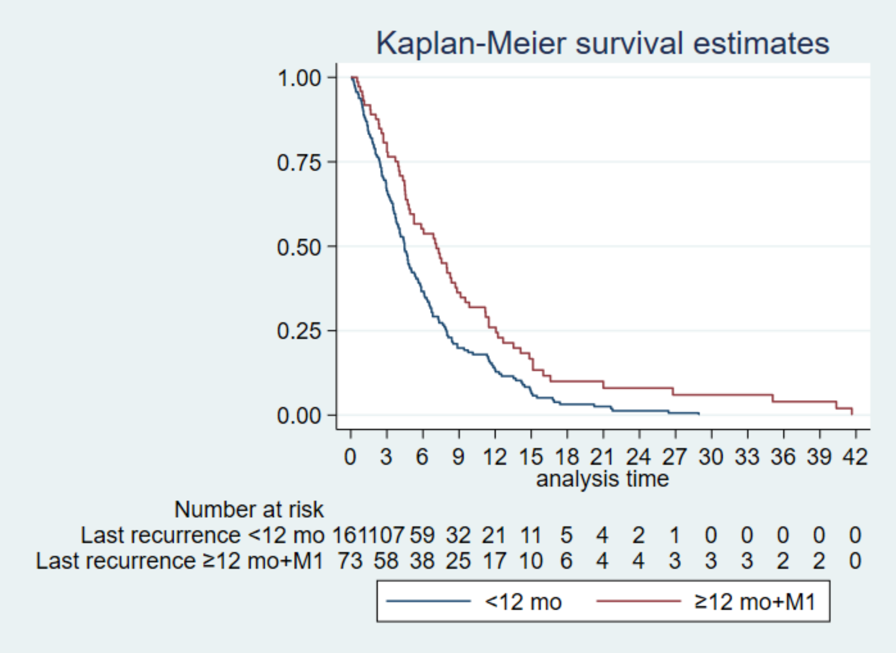

Median OS TTRI <12 months = 4.5 (3.8-5.0) months

Median OS TTRI ≥12 months+M1 = 7.1 (4.8-8.4) months

HR = 0.62 (0.47-0.84), p-value= 0.002
